# Morphological and molecular characterisation of *Tripylina gorganensis* from the Slovak Republic as a contribution to the redescription of the species

**DOI:** 10.21307/jofnem-2021-048

**Published:** 2021-06-23

**Authors:** Marek Renčo, Katarzyna Rybarczyk-Mydłowska, Łukasz Flis, Magdalena Kubicz, Grażyna Winiszewska

**Affiliations:** 1 Institute of Parasitology, Slovak Academy of Sciences, Hlinkova 3, Košice, Slovak Republic; 2 Museum and Institute of Zoology PAS, Wilcza 64, 00-679 Warsaw, Poland

**Keywords:** Redescription, *Fagus sylvatica*, Molecular, Morphology, Morphometrics, Phylogeny, Ribosomal DNA, Taxonomy, *Tripylina gorganensis*

## Abstract

Specimens of *Tripylina gorganensis*, collected from a natural beech forest in Slovak Republic, are described and illustrated. These nematodes were initially identified as an undescribed species, morphologically similar to *Tripylina gorganensis* described from Iran. An important feature distinguishing both species was the presence of post-vulval uterine sac (PUS) in specimens from Slovak Republic, which, according to the original description (Asghari et al., 2012), was absent in *Tripylina gorganensis*. However, a careful re-examination of type specimens performed in this study revealed that *T. gorganensis* also has the post-vulval uterine sac. Consequently, the findings of the morphological and molecular studies performed on the Slovak population and observations on the type material contribute to the redescription of *T. gorganensis*.

The genus *Tripylina* Brzeski, 1963 is represented by free-living, predacious species that inhabit soil, litter, moss, and other semi-wet biotopes (Andrássy, 2007) and are often found in close proximity to various tree species (Asghari et al., 2012; Xu et al., 2013; Zhao, 2009). This genus currently includes 22 species described from different regions of the world (Table 1).

**Table 1.** Alphabetical list of *Tripylina* species and country name from which they were originally described.

Species	Country
*T. arenicola* (de Man, 1880) Brzeski, 1963	The Netherlands
*T. bravoae* Cid del Prado Vera et al., 2012	Mexico
*T. gorganensis* Asghari et al., 2012	Iran
*T. iandrassyi* Cid del Prado-Vera et al., 2016	Mexico
*T. ixayocensis* Cid del Prado Vera et al., 2012	Mexico
*T. kaikoura* Zhao, 2009	New Zealand
*T. longa* Brzeski & Winiszewska-S´lipin´ska, 1993	Italy
*T. macroseta* (Vinciguerra & La Fauci, 1978) Tsalolikhin, 1983	Italy
*T. manurewa* Zhao, 2009	New Zealand
*T. montecilloensis* Cid del Prado Vera et al., 2012	Mexico
*T. puxianensis* Xu et al., 2013	China
*T. rorkabanarum* Cid del Prado-Vera et al., 2016	Mexico
*T. sheri* Brzeski, 1963	USA (California)
*T. stramenti* (Yeates, 1972) Tsalolikhin, 1983	New Zealand
*T. tamaki* Zhao, 2009	New Zealand
*T. tearoha* Zhao, 2009	New Zealand
*T. tlamincasensis* Cid del Prado Vera et al., 2012	Mexico
*T. ursulae* (Argo & Heyns, 1973) Tsalolikhin, 1983	South Africa
*T. valiathani* Tahseen & Nusrat, 2010	India
*T. yeatesi* Zhao, 2009	New Zealand
*T. ymyensis* Tahseen & Nusrat, 2010	China
*T. zhejiangensis* Pham et al., 2013	China

Starting with the work by Zhao (2009), half of the described species have been characterized molecularly by partial sequences of either one or two of the ribosomal DNA genes (18 S and/or 28 S rDNA). The rDNA-based phylogenetic analyses revealed the well-supported monophyletic status of *Tripylina* (Asghari et al., 2012; Cid del Prado-Vera et al., 2012, 2016; Xu et al., 2013; Zhao, 2009). The *Tripylina* clade was shown to be more closely related to the Enoplida representatives like *Pseudocella* Filipjev, 1927, *Deontostoma* Filipjev, 1916 or *Alaimus* de Man, 1880 rather than *Tripyla* Bastian, 1865, *Tripylella* Brzeski & Winiszewska-Ślipińska, 1993 or *Tobrilus* Andrássy, 1959 (Tripylidae, 62 Triplonchida) supporting Andrássy’s (2007) view that *Tripylina* could be subsumed under Enoplida, rather than the Tripylidae de Man, 1876 associated with the Triplonchida (Cid del Prado-Vera et al., 2012, 2016; Zhao, 2009, 2011).

The present work focuses on morphological and molecular characterisation of the *Tripylina gorganensis* found during an ecological study, on the impact of natural disturbance on soil nematode communities’ structures in a natural beech forest (*Fagus sylvatica* L.) in the Slovak Republic. Re-examination of the paratypes of *T. gorganensis* has pointed out the necessity to redescribe this species. *T. gorganensis* happens to be another European species following the reports of *T. arenicola, T. longa*, and *T. macroseta*.

## Material and methods

### Soil sampling, nematode extraction, and processing

#### Soil

In total, ten representative soil samples composed from five subsamples were collected from the five permanent research plots (10 × 10 m) established in the undisturbed natural beech forest in Opátka (48°47’N, 21°04’E, at 776 m a.s.l.), a small village in Košice district, eastern Slovakia. Samples were collected twice, five in May 2016 and five in September 2018, and examined in two laboratories, first in Slovakia and later in Poland.

Each sample (collected in 2016) was homogenized by gentle hand mixing. A total of 100 g of soil was then soaked in 1 l of tap water for 30–60 min and processed by the Cobb sieving and decanting method (Cobb, 1918) followed by the modified Baermann technique (van Benzooijen, 2006). Subsequently, nematodes were extracted from the aqueous soil suspensions using a set of two cotton-propylene filters. Suspensions containing nematodes were collected after 24 h. The nematode suspensions were subsequently examined under a stereomicroscope (40 × and 60 × magnification). After the excess water was removed, five *Tripylina* individuals was set aside for molecular analyses and therefore placed in an Eppendorf tube containing water and subsequently sent to Poland. The remaining nematodes were used to morphological and morphometric study, fixed with a hot 99:1 solution of 4% formaldehyde and pure glycerin (Seinhorst, 1962) and then processed to anhydrous glycerin (Seinhorst, 1959). Permanent slides were made and *Tripylina* nematode individuals were studied using a light microscope Eclipse 90*i* Nikon, Japan at 100, 200, 400, 600, and 1000 × magnification.

The soil samples collected in 2018 were extracted in Poland by the decantation and sieving method followed by the centrifugal flotation method (van Benzooijen, 2006). Extracted nematodes were heat-killed using tap water at 60°C. Some of the selected individuals (as well as a few individuals from 2016 that arrived alive in water from Slovakia) were fixed in TAF and subsequently submitted for morphological analysis. Photographs and measurements were taken from 16 females and 14 males with a Leica DFC 500 camera on a Leica DM 5000B microscope. The remaining nematodes were fixed in DESS and temporary slides were prepared. With photographic documentation collected, the examined specimens were washed with PBS and subsequently with sterile water and then submitted for molecular studies.

### Nematode lysis, amplification, and sequencing of 18 S and 28 rDNA fragments

Ten selected individual nematodes (five collected in 2016 and five in 2018) were transferred to separate 0.2 ml polymerase chain reaction (PCR) tubes containing 25 μl sterile water. An equal volume of the lysis buffer, as described in Holterman et al. (2006), was added. The lysis occurred in a Thermal cycler (Veriti 96*-*Well Thermal Cycler, Applied Biosystems, Foster City, CA, USA) at 65°C for 3 h followed by a 5 min incubation at 100°C. The obtained single nematode lysate (crude DNA extract) was either used immediately as a DNA template for a PCR reaction or stored at ‒20°C.

Nearly full length 18 S rDNA (1,6 kb) was amplified in two overlapping fragments using the following primer combinations: 988 F combined with 1912R and 1813F combined with 2646 R (as in Holterman et al., 2006). The 28 S rDNA (1 kb) sequence was also amplified in two parts using the 61 F primer (Holterman et al., 2006) combined with MCB1R (Dobosz et al., 2013) and the D2A primer combined with D3B (Nunn, 1992). Amplification of the 18 S and 28 S rDNA fragments was performed in reactions containing 12.5 μl Color Perpetual Taq PCR Master Mix (2x) or Color Perpetual OptiTaq PCR Master Mix (2x) (EURx, Gdańsk, Poland), 1 μl of the forward and reverse primer (5 μM each), the 3 μl DNA template and sterile Milli-Q water to 25 μl of a total volume. All PCR reactions were performed in Veriti 96*-*Well Thermal Cycler (Applied Biosystems, Foster City, CA, USA) as follows: an initial denaturation step at 94°C for 3 min, followed by 35–40 cycles at 94°C for 30 s, 54°C (58°C in case of D2A-D3B primer combination) for 30 s and 72°C for 70 s with a final incubation for 5–7 min at 72°C. Amplicons were visualised under UV illumination after Midori Green (Nippon Genetics Europe, Duren, Germany) or Simply Safe (EURx, Gdańsk, Poland) gel staining and gel electrophoresis. Excess dNTPs and unincorporated primers were removed from the PCR product using the Clean-Up Purification Kit (A&A Biotechnology, Gdynia, Poland) or alternatively the enzymatic mixture of Fast Polar-BAP – Thermosensitive Bacterial Alkaline Phosphatase (EURx, Gdańsk, Poland) and Exonuclease I – (EURx, Gdańsk, Poland) was used.

Both the 18 S and 28 S rDNA fragments were successfully amplified and sequenced from all 10 processed nematode individuals. There were no sequence variations, neither in the 18 S rDNA, nor within the 28 S rDNA region. The obtained sequence fragments of 18 S rDNA and 28 S rDNA were deposited in GenBank under the following accession numbers: MK722523 and MK733311.

### Phylogenetic analysis

The newly obtained sequences of *Tripylina* (18 S and 28 S rDNA fragments) were analyzed with publicly available (GenBank) rDNA sequences, using the BioEdit program (v. 7.2.5; Hall, 1999). The 18 S rDNA dataset included the Enoplida sequences from representatives of the genera: *Tripylina*, *Trischistoma*, *Trefusia* de Man, 1893, *Bathylaimus* Cobb, 1894, *Deontostoma*, *Phanoderma* Bastian, 1865, *Pseudocella*, *Thoracostoma* Marion, 1870, *Rhabdolaimus* de Man, 1880, *Syringolaimus* de Man, 1888, *Campydora* Cobb, 1920, *Oxystomina* Filipjev, 1918, and *Ironus macramphis* Schuurmans Stekhoven & Teunissen, 1938 as an outgroup. The species used in the analysis were selected based on the phylogenetic information available in van Megen et al. (2009) and Cid del Prado-Vera et al. (2016). The GenBank sequences that were not unique (did not contain any nucleotide difference as compared to other sequences in the alignment) were excluded from the alignment as uninformative data. For instance, the sequence JQ433064 (*T. ixayocensis*; Cid del Prado-Vera et al., 2012) was not included as this short 18 S rDNA fragment was identical (BLAST 100% identical) to *T. arenicola* (KJ636242, KJ636243), *T. manurewa* (FJ480408) and other sequences from undetermined *Tripylina* species. The 28 S rDNA data set was constructed using representatives of *Tripylina* and *Trischistoma* only. Additionally, a *Deontostoma* sp. sequence was chosen as an outgroup. More distantly related genera, represented already in the 18 S data set, were not included into the 28 S rDNA analysis as they were too variable to align properly.

The final multiple-sequence alignments comprised of 1,705 overlapping characters in case of 18 S rDNA and 676 characters in case of 28 S. Substitution models for both, 18 S and 28 S rDNA data sets were tested using FindModel – an online implementation of the MODELTEST program (Posada and Crandall, 1998).

The Bayesian 18 S rDNA and 28 S rDNA phylogenies were constructed with the program MrBayes v. 3.1 (Ronquist and Huelsenbeck, 2003). Four independent runs were performed with four Markov chains per run in each analysis. The program was run for 1.5 × 10^6^ generations in case of the bigger 18 S rDNA data set and for 400,000 generations in case of the smaller 28 S rDNA data set. The first 150,000 generations were discarded as burn-in in case of the 18 S rDNA analysis and 20,000 generations in case of the 28 S rDNA one. Sample frequency in both cases was 200 generations. The sampled trees were combined in single 50% majority-rule 18 S and 28 S rDNA trees. Stabilization of the likelihood and parameters was checked with the program Tracer (v. 1.6; Rambaut et al., 2014).

## Results

### *Tripylina gorganensis* Asghari et al., 2012

(Figs. 1–3).

**Figure 1:**
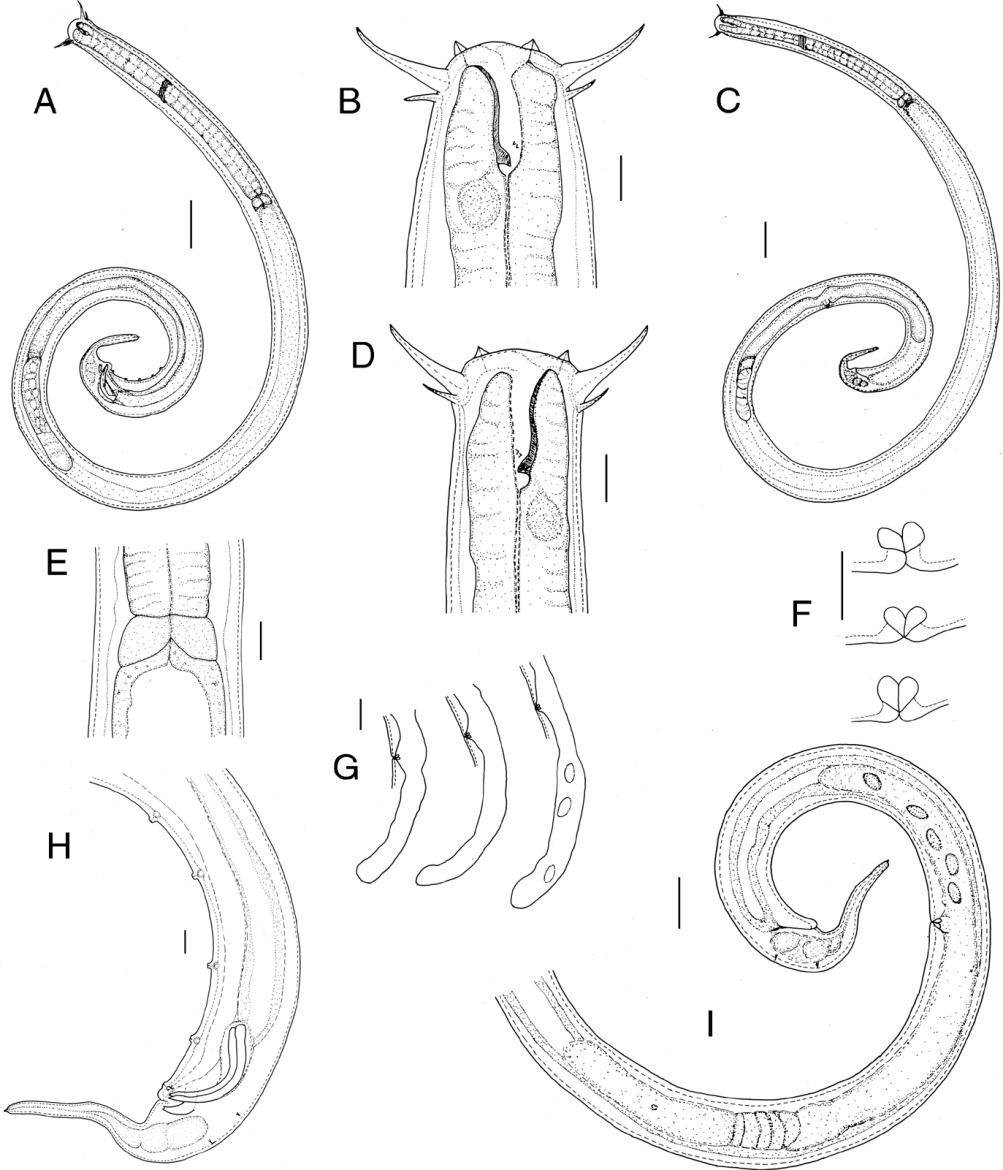
*Tripylina gorganensis* from Slovakia. A: Male entire body; B: Anterior region (female); C: Female entire body; D: Anterior region (male); E: Cardiac region; F: Vaginal sclerotisation; G: Post-vulval uterine sac; H: Male posterior region; I: Female posterior region. (Scale bars: A, C = 50 µm; B, D–F, H = 10 µm; G = 20 µm; I = 30 µm).

**Figure 2:**
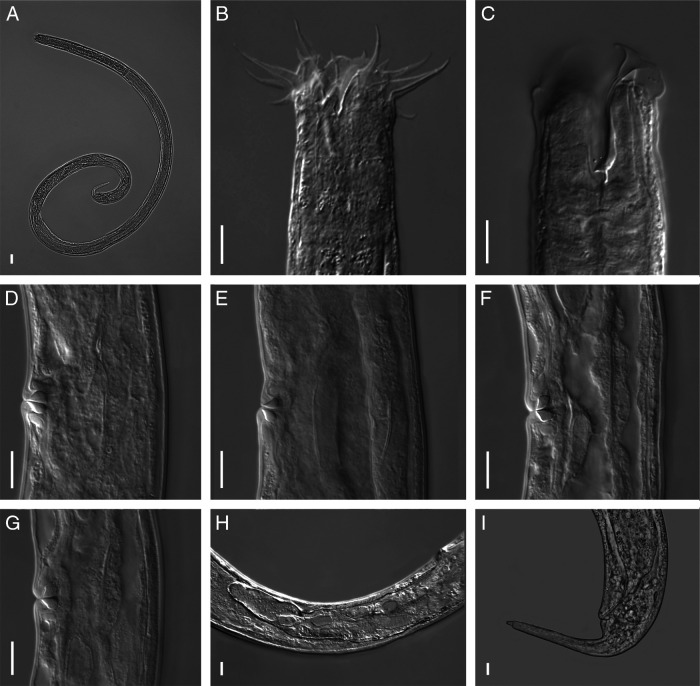
*Tripylina gorganensis* from Slovakia, female. A: Entire body; B, C: Anterior region; D–G: Vaginal sclerotisation; H: Post-vulval uterine sac; I: Posterior region. (Scale bars = 10 µm).

**Figure 3:**
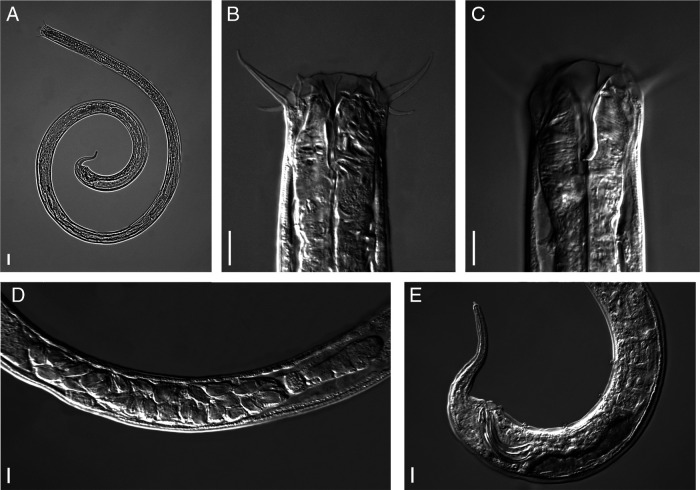
*Tripylina gorganensis* from Slovakia, male. A: Entire body; B, C: Anterior region; D: Testis; E: Posterior region. (Scale bars = 10 µm).

### Measurements

Measurements of the European population of the *Tripylina gorganensis* are given in Table 2.

**Table 2.** Morphometrics of *Tripylina gorganensis* from Iran and Slovakia.

	Iran		Slovakia	
Character	Females	Males	Females	Males
n	4	5	16	14
L	1,813.0 ± 53.9 (1,754–1,860) 2.97	1,675.0 ± 96.0 (1,558–1,790) 5.73	1,694.3 ± 173.1 (1,472–2,038) 10.22	1,662.2 ± 230.8 (1,358–2,017) 13.89
a	52.6 ± 1.5 (51.6–54.7) 2.85	52.5 ± 4.9 (45.7–59.7) 9.33	41.0 ± 5.6 (34.4–51.5) 13.55	42.2 ± 3.6 (35.8–48.5) 8.45
b	6.1 ± 0.1 (5.9–6.2) 1.64	5.6 ± 0.3 (5.2–5.6) 5.36	5.4 ± 0.3 (4.9–5.8) 6.12	5.3 ± 0.4 (4.7–6.0) 7.71
c	32.1 ± 3.2 (29.1–35.8) 9.97	33.6 ± 6.5 (23.9–39.8) 19.35	20.0 ± 1.8 (18.1–25.0) 8.88	19.0 ± 1.9 (15.0–22.4) 9.97
c’	2.3 ± 0.3 (2.0–2.6) 13.01	1.9 ± 0.3 (1.6–2.5) 15.79	2.8 ± 0.4 (2.1–3.4) 12.62	2.6 ± 0.2 (2.3–3.1) 7.36
V (%)	79.6 ± 1.2 (78.4–81.2) 1.51		77.0 ± 1.1 (74.6–78.7) 1.47	
Lip region width	23.3 ± 2.6 (21–26) 11.16	23.8 ± 0.8 (23–25) 3.36	29.2 ± 1.3 (27.4–33.0) 4.51	29.3 ± 2.3 (25.5–33.3) 7.88
Maximum body width	34.5 ± 1.0 (34–36) 2.90	32.0 ± 2.1 (30–35) 6.56	41.6 ± 3.5 (36.3–47.8) 8.48	39.3 ± 3.6 (34.7–45.3) 9.04
Anal body width	24.5 ± 1.9 (23–27) 7.76	26.6 ± 1.1 (25–28) 4.14	30.8 ± 3.7 (25.9–35.9) 11.96	34.2 ± 3.1 (29.5–39.9) 9.14
Dorsal tooth from anterior body end			27.7 ± 0.9 (25.9–29.0) 3.29	27.5 ± 1.1 (25.1–29.3) 4.00
Nerve ring from anterior end			124.9 ± 9.4 (106–142) 7.54	124.6 ± 13.5 (112–149) 10.85
Pharynx length	297.0 ± 6.8 (288–304) 2.29	297.0 ± 7.6 (286–307) 2.56	316.5 ± 29.0 (256–365) 9.18	315,7 ± 38.5 (256–369) 12.21
Pharynx (% of body length)			18.7 ± 1.1 (17.1–20.2) 6.11	19.1 ± 1.5 (16.7–21.5) 7.82
Head to vulva	1442.0 ± 58.0 (1392–1507) 4.02		1304.9 ± 132.7 (1136–1598) 10.17	
Vulva to anus	313.0 ± 13.1 (295–325) 4.19		303.3 ± 44.9 (255–407) 14.81	
Tail length	57.0 ± 6.5 (49–64) 11.40	51.2 ± 9.2 (45–67) 17.97	84.8 ± 5.7 (75.7–94.8) 6.73	87.4 ± 7.6 (75.7–99.5) 8.67
Tail (% of body length)			5.0 ± 0.4 (4.0–5.5) 7.94	5.3 ± 0.6 (4.5–6.7) 10.40
Rectum/cloaca length			31.0 ± 2.2 (26.8–34.2) 7.21	42.0 ± 4.9 (34.0–49.2) 11.62
Spineret length			2.1 ± 0.2 (1.8–2.3) 7.86	2.3 ± 0.4 (1.7–2.9) 17.20

Note: All measurements are in µm and in the form: mean ± S.D. (range) CV.

### Description

#### Female

Body long and relatively slender, arcuate ventrally after gentle heat treatment with posterior part of body more curved than anterior. Cuticle smooth, 1.8 (1.4–2.3) µm thick. Body pores small and numerous, distributed along entire body. Pseudocoelomocyte cells 14.7 (10.2–20.5) µm wide and 27.2 (15.1–41.6) µm long, located along body, their number varying from one to seven pairs. Max. body diam. generally recorded at ovary level, occasionally at level of vulva. Head rounded, 29.3 (27.4–33.0) µm diam., smooth, continuous with body contour. Inner labial papillae conical, 2.2 (1.7–2.4) µm long. Six outer labial setae and four short cephalic setae arranged in a single whorl. Outer labial setae strongly developed, 21.2 (17.0–24.2) µm long, more or less arcuate, bent at tip and directed anteriorly, four cephalic setae 9.1 (7.1–12.4) µm long, thinner than outer labial setae. Stoma 31.6 (29.6–34.9) µm long, funnel-shaped with weakly sclerotised cheilostom, rest of stoma with dorsal wall thickened almost for its entire length, thickening gradually expanding from beginning of mouth to dorsal tooth, where wall thickness is largest. Dorsal tooth relatively large, triangular, directed posteriad, lying 28 (26–29.0) µm from anterior end of body. Two small subventral teeth 3.1 (2.3–3.7) µm anterior to dorsal tooth. Amphids cup-shaped, located 19.0 (17.4–20.6) µm from anterior extremity. Excretory pore 148 (133–175) µm from anterior end. Cervical region with three ventromedian setae. One female with two lateral setae lying 54 and 138 µm from anterior end of body. Pharynx cylindrical and muscular, slightly enlarged anteriorly to surround stoma. Dorsal pharyngeal gland opening at level of dorsal tooth. Nerve ring located at 39.6 (35.5–47.0)% of pharyngeal length from anterior end. Excretory pore not observed. Cardia located between pharynx and intestine, relatively large and wide with gland-like bodies. In intestine of several individuals an ingested nematode was observed. Rectum arcuate, 31.0 (26.8–34.2) µm long. Tail with poorly visible caudal glands, terminated by a small, tubular spinneret 2.1 (1.8–2.3) µm long. Two subdorsal caudal setae situated a short distance anterior and posterior to anus. Reproductive system 374 (224–726) µm long, monodelphic-prodelphic with post-vulval uterine sac 145 (103–190) µm long, occasionally containing large, oval sperm. *Pars refringens vaginae* clearly separated, tear-shaped, sometimes almost semi-spherical sclerotisations (4.8–6.6 µm long and 5.4–7.6 µm wide). *Pars distalis vaginae* clearly marked, thick walled. Vulva with protuberant lips.

#### Male

Morphology and morphometrics similar to that of female. Genital branch 668 (312–849) µm long. Testis outstretched, filled with oval-shaped spermatozoa. Spicule narrow with arched proximal and acute distal end, 54 (48–59) µm long, evenly tapering, sickle-shaped. Gubernaculum well developed, 10.1 (9.0–11.8) µm long, bifurcate proximally, surrounding spicules dorsally and laterally. Cloaca 42.0 (34.0–49.2) µm long. Ventromedian, precloacal supplements five in number, papillate, irregularly arranged. Distance between first and last supplement 119 (77–159) µm. First supplement lying 8.3–11.9 µm from cloacal aperture, second 28–41, third 45–83, fourth 73–132, and fifth 96–167 µm.

#### Material examined

Iran. Soil and litter, at a depth of 0–15 cm under a hawthorn tree (*Crataegus monogyna* L.) in a forest, Naharkhoran region, Gorgan, Golestan Province, northern Iran. One paratype female and two paratype males deposited at the National Nematode Collection, New Zealand (NNCNZ).

Slovak Republic. Soil and litter, at a depth of 0–20 cm under a beech tree (*Fagus sylvatica* L.) in a natural forest in Opátka (N 48°47’, E 21°04’, 776 m a.s.l.), a small village in the Košice district, eastern Slovakia. The description is based on the morphology and morphometry of 16 females and 14 males.

Voucher specimens were deposited in the National Nematode Collection in New Zealand (NNCNZ), the Crown Research Institute Landcare Research New Zealand Ltd, New Zealand (2 females, 2 males) and in the nematode collection at the U.S.D.A, Beltsville, USA (one female, one male). The remaining specimens were deposited in the Institute of Parasitology, Slovak Academy of Sciences, Slovak Republic (4 females, 4 males) and in the nematode collection at the Museum and Institute of Zoology, PAS, Warsaw, Poland (9 females, 7 males).

#### Remark

Specimens collected from a natural beech forest in Slovak Republic were first identified as an undescribed species, morphologically similar to *Tripylina gorganensis* described from Iran (Asghari et al., 2012). An important feature distinguishing both species was the presence of post-vulval uterine sac (PUS) in specimens from Slovakia, which, according to the original description (Asghari et al., 2012), was absent in *Tripylina gorganensis*. A careful re-examination of type specimens revealed that *T. gorganensis* also has a post-vulval uterine sac. As a consequence a redescription of *T. gorganensis* was needed. Morphologically, the specimens from Slovak Republic agree well with the paratypes of *T. gorganensis*. The morphometrics of the beech forest nematodes agrees with most of the dimensions in the original description except for some minor differences: they have a longer tail (76–95 vs 49–64 µm in females, 76–99 vs 45–67 µm in males) and a lower value of the coefficient c (18–25 vs 27–36 in females and 15–22 vs 24–40 in males). We consider these differences may be a part of the intraspecific variation within this species.

#### Diagnosis and relationships

*T. gorganensis* is characterized by having a long body, dorsal wall thickened almost over the entire length of stoma, a large dorsal tooth directed posteriad, subventral denticles located anterior to dorsal tooth, three ventromedian setae and two pairs of lateral cervical setae in the cervical region, a relatively long post-vulval uterine sac, two subdorsal caudal setae situated a short distance anterior and posterior to anus and male having five to six papillate ventromedian supplements.

**Figure 4:**
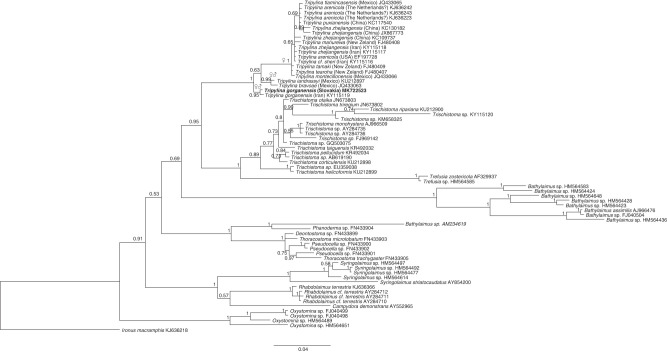
18 S rDNA-based Bayesian phylogeny of the Trischistomatidae and some other representatives of Enoplida. The new population of *Tripylina gorganensis* is indicated in bold. Numbers near nodes indicate posterior probabilities. ♀♂ = all species from the marked clade are dioecious; ♀ = males of the species from this clade are unknown or extremely rare.

**Figure 5:**
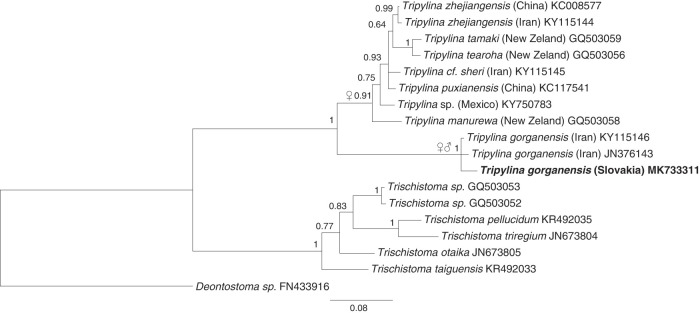
28 S rDNA-based Bayesian phylogeny of the Trischistomatidae. The new population of *Tripylina gorganensis* is indicated in bold. Numbers near nodes indicate posterior probabilities. ♀♂ = all species from the marked clade are dioecious; ♀ = males of the species from this clade are unknown or extremely rare.

*Tripylina gorganensis* together with *T. bravoae*, *T. iandrassyi* and *T. longa* belongs to a small group of *Tripylina* whose common, distinctive features are: stoma with dorsal wall thickened almost on its entire length, the thickening gradually expands from the beginning of the mouth to the dorsal tooth, a large dorsal tooth directed posteriorly and the presence of males. Their close relationship, except *T. longa* for which ribosomal sequences are not available, is supported by the molecular data (Fig. 4). Phylogenetic analyses confirm the basal positioning of *Tripylina gorganensis* (Asghari et al., 2012) within a well supported *Tripylina* clade (Figs 4 and 5). BLAST analysis revealed the sequence identity between *T. gorganensis* populations from Iran and Slovakia of more than 99% (4 bp difference) in case of 18 S rDNA and of 97–98% (16–20 SNPs) in case of 28 rDNA.

**Key to species of the** ***Tripylina longa*** **group**

**Table:** 

1. Subventral denticles located posterior to dorsal tooth	*T. iandrassyi*
Subventral denticles located anterior to dorsal tooth	2
2. Vaginal sclerotisations oval, female without ventromedian setae in cervical region, sparse somatic setae present	*T. bravoae*
Vaginal sclerotisations tear-shaped to semi-spherical, female with ventromedian setae in cervical region, sparse somatic setae absent	3
3. Pharynx = 256–369 µm, b = 4.7–6.2	*T. gorganensis*
Pharynx = 216–242 µm, b = 6.3–7.4	*T. longa*
